# Thermo-Responsive Nanocomposite Hydrogels Based on PEG-*b*-PLGA Diblock Copolymer and Laponite

**DOI:** 10.3390/polym11020250

**Published:** 2019-02-02

**Authors:** Tomoki Maeda, Midori Kitagawa, Atsushi Hotta, Satoshi Koizumi

**Affiliations:** 1 Frontier Research Center for Applied Atomic Sciences, Ibaraki University, 162-1 Shirakata, Tokai, Ibaraki 319-1106, Japan; satoshi.koizumi.prof@vc.ibaraki.ac.jp; 2 Department of Mechanical Engineering, Keio University, 3-14-1, Hiyoshi, Kohoku-ku, Yokohama 223-8522, Japan; midorikitagawa.10@gmail.com (M.K.); hotta@mech.keio.ac.jp (A.H.)

**Keywords:** PEG, PLGA, nanocomposite, thermo-responsive hydrogel

## Abstract

Poly(ethylene glycol)-*b*-poly(d,l-lactide-*co*-glycolide) (PEG-*b*-PLGA) diblock copolymers are widely known as polymeric surfactants for biomedical applications, and exhibit high solubility in water compared to PLGA-*b*-PEG-*b*-PLGA triblock copolymers known as gelation agents. In order to overcome the difficulties in the preparation of thermo-responsive hydrogels based on PLGA-*b*-PEG-*b*-PLGA due to the low solubility in water, the fabrication of thermo-responsive hydrogels based on PEG-*b*-PLGA with high solubility in water was attempted by adding laponite to the PEG-*b*-PLGA solution. In detail, PEG-*b*-PLGA with high solubility in water (i.e., high PEG/PLGA ratio) were synthesized. Then, the nanocomposite solution based on PEG-*b*-PLGA and laponite (laponite/PEG-*b*-PLGA nanocomposite) was fabricated by mixing the PEG-*b*-PLGA solutions and the laponite suspensions. By using the test tube inversion method and dynamic mechanical analysis (DMA), it was found that thermo-responsive hydrogels could be obtained by using PEG-*b*-PLGA, generally known as polymeric surfactants, and that the gelation temperature was around the physiological temperature and could be regulated by changing the solution composition. Furthermore, from the structural analysis by small angle neutron scattering (SANS), PEG-*b*-PLGA was confirmed to be on the surface of the laponite platelets, and the thermosensitive PEG-*b*-PLGA on the laponite surface could trigger the thermo-responsive connection of the preformed laponite network.

## 1. Introduction

Block copolymers with hydrophilic poly(ethylene glycol) (PEG) and hydrophobic poly(d,l-lactide*-co-*glycolide) (PLGA) such as PEG-*b*-PLGA, PLGA-*b*-PEG-*b*-PLGA, and PEG-*b*-PLGA-*b*-PEG are known as the promising block copolymers for biomedical applications. The hydrophilic PEG block introduces the biocompatibility to the block copolymers, and the hydrophobic PLGA block composed of lactide (LA) and glycolide (GA) introduces the biodegradability to the block copolymers because of the ester backbones [1]. In addition, PEG and PLGA have already been approved by the Food and Drug Administration (FDA). In terms of the molecular structures, PEG-*b*-PLGA diblock copolymers are generally used as polymeric surfactants or nanoparticles for drug delivery. The diblock copolymers possess high solubility in water but cannot form hydrogels. On the other hand, PLGA-*b*-PEG-*b*-PLGA and PEG-*b*-PLGA-*b*-PEG triblock copolymers are generally used as thermo-responsive hydrogels for drug delivery systems and injectable cell scaffold matrixes [2,3,4,5,6,7,8]. However, the triblock copolymers are less water soluble.

In particular to thermo-responsive hydrogels based on the triblock copolymers, the less water-soluble property is important but the low solubility of the triblock copolymers in water causes the difficulties in the preparation process. The most important factor influencing the properties and the applications of amphiphilic block copolymers are known to be the nature and the length of the hydrophobic segment. For example, the triblock copolymers with high PLGA content becomes less water soluble but can swell in water (i.e., can form hydrogels). In fact, Shim et al. [4] reported that it took 2–4 days at 0 °C to dissolve PLGA-*b*-PEG-*b*-PLGA in water due to the high PLGA content. Shim et al. [4] also reported that the molecular weight ratio between the PEG and the PLGA blocks (PEG/PLGA ratio) in the PLGA-PEG-PLGA copolymers should be 0.56 or lower in order to obtain the thermo-responsive hydrogels [4]. Therefore, in order to fabricate hydrogels using highly water-soluble polymers, a different approach was thought to be required.

The nanocomposite approach is known as a facile approach to obtain hydrogels with high functionalities (i.e., mechanical toughness and stimuli-responsive properties) arising from the combination of polymers and nanofillers. In particular, as the examples of previous studies using amphiphilic block copolymers and laponite, Sun et al. [9] reported the thermo-responsive gelation of the aqueous composite solution based on Pluronic F127 and laponite around 70 °C (>>physiological temperature) at low solute concentration. Sun et al. [10] also reported the light-activated gelation of the composites based on Pluronic F127, laponite, and photoacid generators. Instead of laponite, the previous studies using amphiphilic block copolymers and graphene nanosheets were also reported. For example, Zu et al. [11] reported the supramolecular hydrogels based on Pluronic F68, graphene nanosheets, and cyclodextrin. According to the previous studies achieving the drastic change in the functionalities, the nanocomposite approach could be a crucial clue to fabricate hydrogels using highly water-soluble polymers, for example, the PEG-*b*-PLGA diblock copolymers with high solubility in water.

In this paper, we focused on PEG-*b*-PLGA diblock copolymers with high PEG/PLGA ratio (i.e., with high solubility in water) and attempted to obtain the thermo-responsive hydrogels with PEG-*b*-PLGA diblock copolymers through the nanocomposite approach [12]. This is because PEG-*b*-PLGA diblock copolymers are polymeric surfactants and exhibit high solubility to water compared to the PLGA-PEG-PLGA triblock copolymers and could overcome the difficulties in the preparation process of thermo-responsive hydrogels based on the triblock copolymers with low solubility in water. In detail, we synthesized PEG-*b*-PLGA diblock copolymers with high PEG/PLGA ratio, and prepared the nanocomposite with PEG-*b*-PLGA diblock copolymer and laponite (laponite/PEG-*b*-PLGA nanocomposite). Laponite is a synthetic magnesium–silicate platelet with negative charges on its plane surface, readily dispersed in water to form clear transparent dispersion [13,14]. We varied the PLGA molecular weight of PEG–PLGA (0.8 and 1.6 k) and the solution composition (i.e., the PEG–PLGA concentration and the laponite concentration). The thermo-responsive gelation behavior was investigated by the test-tube inversion method and by the dynamic mechanical analysis (DMA). Furthermore, the dispersion state of PEG-*b*-PLGA diblock copolymers in the nanocomposite was analyzed by the small angle neutron scattering (SANS) measurements. As a result, it was found that the thermo-responsive hydrogels could be obtained by using PEG-*b*-PLGA with high solubility in water (PEG/PLGA ratio of ~1.3). It was also found that the sol-gel transition temperature (*T*_gel_) were confirmed to be around the physiological temperature (25–37 °C).

## 2. Materials and Methods

### 2.1. Materials

Poly(ethylene glycol) methyl ether (PEG) (*M*_n_ = 1000 g/mol) was purchased from Tokyo Chemical Industry Co. Ltd. (Tokyo, Japan). Glycolide, d,l-lactide, and tin(II) 2-ethylhexanoate were purchased from Sigma–Aldrich Japan (Tokyo, Japan). Laponite XLG^®^ was supplied by BYK Japan KK (Tokyo, Japan). The ultrapure water was prepared through Smart2Pure 3 UV (Thermo Fisher Scientific K.K., Tokyo, Japan) in our laboratory.

### 2.2. Synthesis and Characterization of PEG-b-PLGA

**Figure 1:**
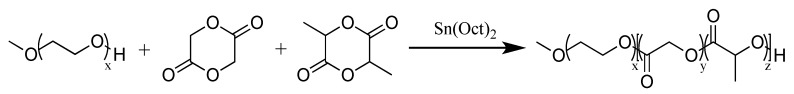
Synthesis of PEG-*b*-PLGA diblock copolymer.

PEG-*b*-PLGA diblock copolymers with different PLGA molecular weight were synthesized from PEG (1000 g/mol) by a ring-opening polymerization as described in a previous paper (Figure 1) [1,15]. In detail, PEG, LA, and GA were put into a Schlenk flask and dried under vacuum overnight. Then, tin(II) 2-ethylhexanoate (0.2 wt %) was added to the Schlenk flask. The flask purged with Ar was then immersed in an oil bath at 155 °C and stirred for 6 h. After completing the reaction, the temperature of an oil bath was reduced to 150 °C. At the same time, in order to remove unreacted monomers, the flask was kept under vacuum for 30 min. The products were dissolved into chloroform and precipitated in diethyl ether. The conversion was confirmed to be ~85%.

The molecular weight and the composition of the synthesized PEG-*b*-PLGA diblock copolymers were determined by the ^1^H-NMR measurements and the gel permeation chromatography (GPC) measurements in the same manner as described in previous studies [16,17,18,19,20,21]. As the sample for the ^1^H-NMR measurements, the PEG-*b*-PLGA diblock copolymer was dissolved into CDCl_3_ and the NMR spectrum was obtained using a 400 MHz NMR spectrometer (AVANCE III 400, Bruker Japan K.K., Yokohama, Japan). The GPC measurements were performed using a high-performance liquid chromatography (HPLC) (Alliance HPLC system, Nihon Waters K.K., Tokyo, Japan). Tetrahydrofuran was used as the eluent at a flow rate of 0.8 mL/min, and polystyrene standards (InfinityLab EasiVial PS-L, Agilent Technologies Japan, Ltd., Tokyo, Japan) were used as the calibration samples for the HPLC.

### 2.3. Preparation of the Aqueous Laponite/PEG-b-PLGA Nanocomposite

The laponite/PEG-*b*-PLGA nanocomposites were fabricated by blending the PEG-*b*-PLGA solution and the laponite suspension. First, the PEG-*b*-PLGA solution and the laponite suspension were prepared separately. The PEG-*b*-PLGA was dissolved in pure water. Laponite was dispersed in pure water, and the suspension was then processed through the autoclave treatment. Eventually, the PEG-*b*-PLGA solutions (2.0, 4.0, 6.0, 8.0, and 10.0 wt %) and the laponite suspension (1.5, 2.0, 2.5 wt %) were prepared, respectively. Then, the PEG-*b*-PLGA solution and the laponite suspension were blended in equal quantity, and a series of laponite/PEG-*b*-PLGA nanocomposites were obtained. Eventually, the nanocomposite solutions with different combinations of laponite (0.75, 1.0, and 1.25 wt %) and PEG-*b*-PLGA (1.0, 2.0, 3.0, 4.0, and 5.0 wt %) were prepared.

### 2.4. Analysis of Thermo-Responsive Gelation

The thermo-responsive gelation behavior of the laponite/PEG-*b*-PLGA nanocomposite specimens were analyzed by the test-tube inversion method and by dynamic mechanical analysis (DMA). The test-tube inversion method is the way to determine the sol or gel state by examining the flow or non-flow behavior of the samples in an inversed test tube. The 0.3 mL of the nanocomposites were sealed into 2 mL vials, and the vials were immersed in a temperature-regulated water bath for 2 minutes. The temperature was controlled by 1 °C from 10 to 60 °C. Specimens were determined as gel if no visual liquid flow was observed 30 s after a vial was inversed. The results were found to be actually reproducible within ±2 °C.

The thermo-responsive gelation of the laponite/PEG-*b*-PLGA nanocomposites was also quantitatively analyzed by DMA using a strain-controlled rheometer (ARES-G2, TA Instruments Japan Inc., Tokyo, Japan). A parallel plate and the Peltier plate of 50 mm in diameter were used for the measurement, and the gap between the plates was kept constant at 0.40 mm. Depending on the linear region of the specimens, the amplitude of strain was adjusted. The oscillatory frequency was set at 10 rad/s. The state of the specimen was analyzed as the gel state when *G*′ is larger than *G*″. The gelation temperature was defined as the crossover point of *G*′ and *G*″.

### 2.5. Microstructural Analysis of PEG-b-PLGA Diblock Copolymers in the Nanocomposite

The microstructure of PEG-*b*-PLGA diblock copolymers in the nanocomposite was determined by the small angle neutron scattering (SANS) measurements. Small angle neutron scattering measurements were performed at BL-20 (iMATERIA) in the Materials and Life Science Experimental Faculty (MLF), Japan Proton Accelerator Research Complex (J-PARC), Tokai, Japan. In the preparation of the samples for SANS measurements, the D_2_O/H_2_O mixture at the D_2_O fraction of 66% was used in order to ignore the scattering from laponite and evaluate only the scattering from PEG-*b*-PLGA. Prior to the contrast-matching SANS experiment, the D_2_O fraction was varied from 0 to 100%, and the contrast matching-point for laponite in water was confirmed to be at the D_2_O fraction of 66%. The result was consistent with the results reported in the previous paper [22]. After the preparation of the aqueous laponite/PEG-*b*-PLGA nanocomposites using the D_2_O/H_2_O mixture at the D_2_O fraction of 66%, the laponite/PEG-*b*-PLGA nanocomposites in the sol state were put into a quartz cell (20 mm in the diameter and 1 mm in the thickness) by a syringe. Then, by heating the quartz cell, the laponite/PEG-*b*-PLGA nanocomposites in the gel state were prepared inside the quartz cell. We also prepared the single-body PEG-*b*-PLGA solution as a reference. For the single-body solution, D_2_O was used instead of H_2_O because good contrast and low background could be obtained [23,24].

The sample in the quartz cell was irradiated with the white neutron beam, and the scattered beam were detected with 3He-sensitive detectors. After the circular average of the distribution of the scattered beam on the detector, the data were merged with respect to the scattering vector *Q* (*Q* = 4πsinθ/λ, where 2θ is the scattering angle and λ is the wavelength of neutron). The obtained scattering profiles were then normalized to the absolute intensity by the glassy carbon standard.

## 3. Results

### 3.1. Characteristics of the Synthesized PEG-b-PLGA Diblock Copolymers

**Figure 2:**
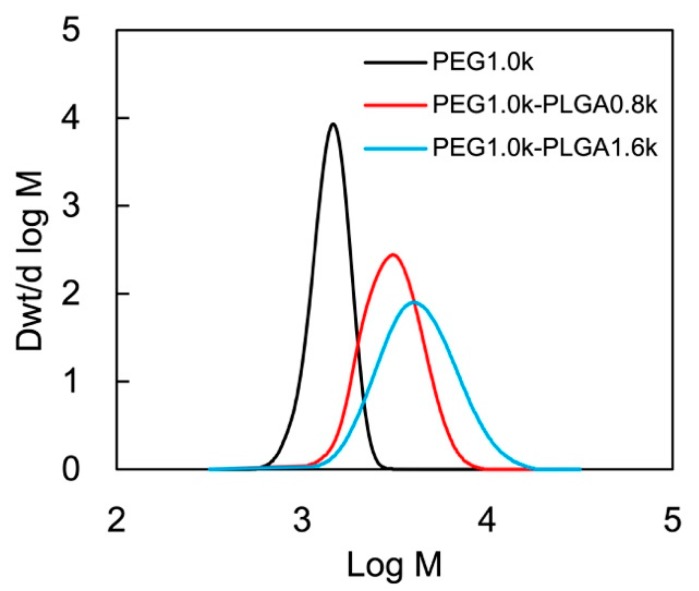
Molecular weight distributions of PEG and the synthesized PEG-*b*-PLGA diblock copolymers determined by GPC.

The molecular weight of PLGA, the PEG/PLGA ratio, and the molar ratio of lactide (LA) and glycolide (GA) (LA/GA ratio) in the PLGA block were analyzed from the ^1^H-NMR spectra and were summarized in Table 1. There was correspondence between the chemical shifts δ and the chemical structures as follows: δ 1.55 as (–OCH(C*H_3_*)CO–), δ 3.38 as (–OC*H_3_*), δ 3.60 as (–OC*H_2_*CH_2_–), δ 4.30 as (–OCH_2_C*H_2_*OCOCH_2_O–), δ 4.80 as (–OC*H_2_*CO–), and δ 5.20 as (–OC*H*(CH_3_)CO–). The molecular weight distribution of the synthesized PEG-*b*-PLGA was analyzed by GPC as shown in Figure 2, and the molecular weight of PEG-*b*-PLGA and the polydispersity (*M*_w_/*M*_n_) were summarized in Table 1. As shown in Figure 2, the molecular weight distributions of the synthesized PEG-b-PLGA diblock copolymers were monomodal. Therefore, it was confirmed that PEG-*b*-PLGA diblock copolymers with high PEG/PLGA ratio were successfully synthesized.

### 3.2. Thermo-Responsive Gelation of the Laponite/PEG-b-PLGA Nanocomposites

**Figure 3:**
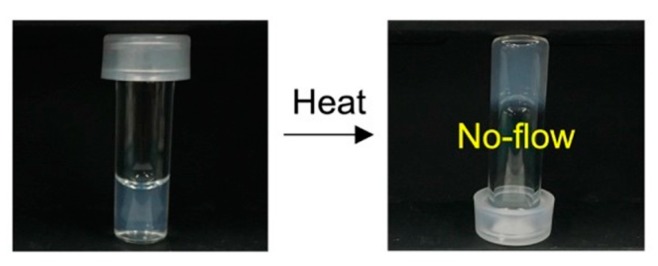
The sol and the gel states of the aqueous laponite/PEG-PLGA nanocomposite with 0.75 wt % of laponite and 3.0 wt % of PEG1.0k–PLGA1.6k.

The sol and the gel states of the laponite/PEG-*b*-PLGA nanocomposite are shown in Figure 3. The nanocomposite displayed here is composed of 0.75 wt % of laponite and 3.0 wt % of PEG1.0k–PLGA1.6k. As shown in Figure 3, it was found that the laponite/PEG-*b*-PLGA nanocomposite responded to the temperature increase and exhibited the non-flow state at higher temperature. It should be noted that the PEG-*b*-PLGA solutions without laponite (i.e., the single-body PEG-*b*-PLGA solutions) were in the flow state (i.e., sol) regardless of the increase in temperature. Therefore, it was found that the thermo-responsive hydrogels could be obtained by using PEG-*b*-PLGA diblock copolymers with high solubility in water.

**Figure 4:**
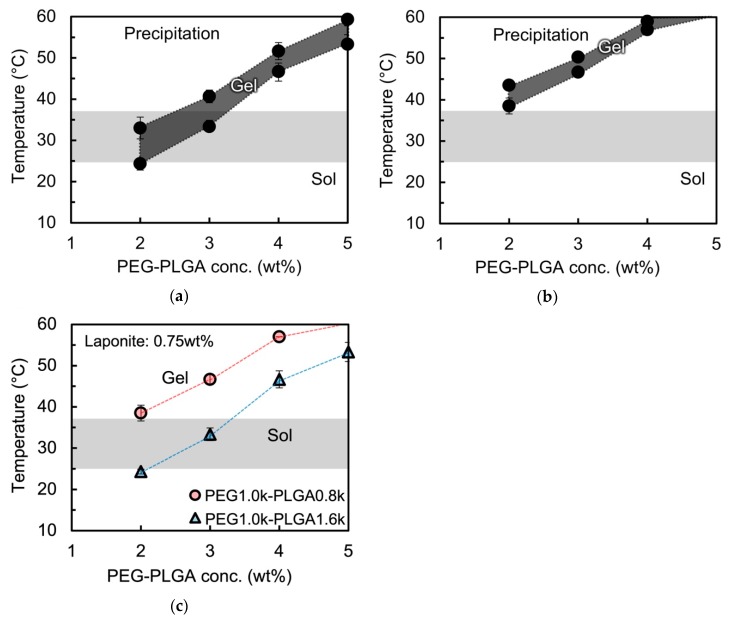
Thermo-responsive gelation behavior of the aqueous laponite/PEG–PLGA nanocomposites with (**a**) PEG1.0k–PLGA1.6k and (**b**) PEG1.0k–PLGA0.8k. The gelation temperatures (*T*_gel_) observed through all the different PLGA molecular weights were plotted in (**c**).

Then, the thermo-responsive gelation behavior of the laponite/PEG-*b*-PLGA nanocomposites were systematically analyzed by varying the PLGA molecular weight (800 and 1600 g/mol) of PEG-*b*-PLGA and the solute composition (i.e. the PEG-*b*-PLGA concentration and the laponite concentration). The laponite concentration was fixed at 0.75 wt % in these experiments. Figure 4a shows the thermo-responsive gelation behavior of the laponite/PEG1.0k-PLGA1.6k nanocomposites. It was found that the nanocomposites exhibited the thermo-responsive gelation behavior at the PEG1.0k–PLGA1.6k concentration of 2 wt % or higher, while the nanocomposites did not present the gel state at the PEG1.0k–PLGA1.6k concentration of 1.0 wt %. It was also found that the gelation temperature increased as the PEG1.0k–PLGA1.6k concentration increased.

In order to enhance the solubility of PEG-*b*-PLGA in water, we decreased the PLGA molecular weight from 1600 to 800 g/mol and evaluated the thermo-responsive gelation behavior of the laponite/PEG-*b*-PLGA nanocomposites. Figure 4b shows the thermo-responsive gelation behavior of the laponite/PEG1.0k–PLGA0.8k nanocomposites. Even when the PLGA molecular weight decreased from 1600 down to 800 g/mol (i.e., the PEG/PLGA ratio increased from ~0.6 up to ~1.3), the critical concentration for gelation and the concentration dependency of the gelation temperature mentioned above were in good agreement with those of the laponote/PEG1.0k–PLGA0.8k nanocomposite.

Figure 4c shows the summary of the thermo-responsive gelation temperature (*T*_gel_) of the nanocomposites using PEG-*b*-PLGA with different PLGA molecular weights. It was clearly revealed that *T*_gel_ increased with the decrease of the PLGA molecular weight. It was also confirmed that the laponite/PEG nanocomposites (i.e., the nanocomposite with PEG homopolymer instead of PEG-*b*-PLGA diblock copolymer) presented no gelation. All these results suggested that the addition of laponite realized the thermo-responsive gelation of PEG-*b*-PLGA with high PEG/PLGA ratio, and that the existence of PLGA hydrophobic block was a key to achieve the thermo-responsive gelation.

**Figure 5:**
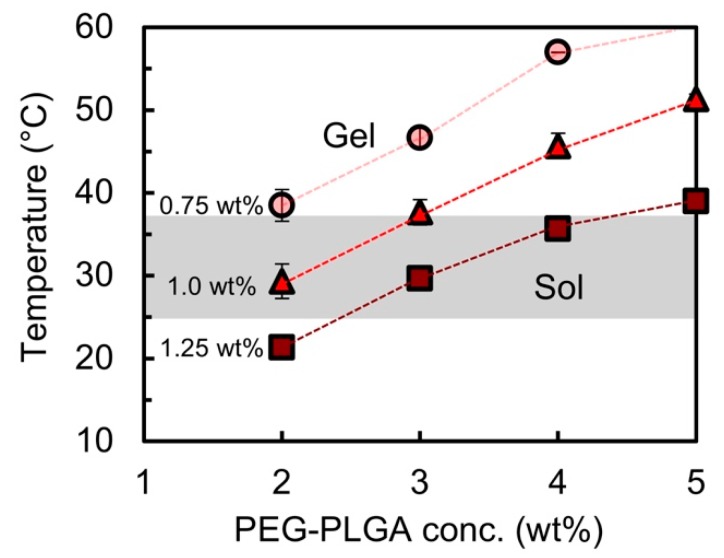
The gelation temperatures *T*_gel_ of the aqueous laponite/PEG1.0k–PLGA0.8k nanocomposites with different laponite concentrations (0.75 wt %: circles, 1.0 wt %: triangles, 1.25 wt %: squares).

In order to regulate the *T*_gel_ of the laponite/ PEG1.0k-PLGA0.8k nanocomposites to be around the physiological temperature, the laponite concentration was varied from 0.75 to 1.25 wt %. Figure 5 shows the thermo-responsive gelation behavior of the laponite/PEG1.0k–PLGA0.8k nanocomposites with different laponite concentrations. It was found that the critical laponite concentration to initiate the thermo-responsive gelation was 0.75 wt %, and that the *T*_gel_ decreased as the laponite concentration increased. In other words, the nanocomposites did not exhibit the thermo-responsive gelation behavior at the laponite concentration of 0.50 wt %. Additionally, regardless of the laponite concentration (0.75–1.25 wt %), it was confirmed that the *T*_gel_ increased as the PEG-*b*-PLGA concentration increased, and that the critical concentration of PEG-*b*-PLGA in the nanocomposite for the gelation was 2.0 wt %. In other words, the nanocomposites did not present the thermo-responsive gelation behavior at the PEG-*b*-PLGA concentration of 1.0 wt %. 

Hereafter, we mainly selected the PEG1.0k–PLGA0.8k for the further measurement because the PEG1.0k–PLGA0.8k was the PEG-*b*-PLGA diblock copolymer with highest PEG/PLGA ratio in this paper.

**Figure 6:**
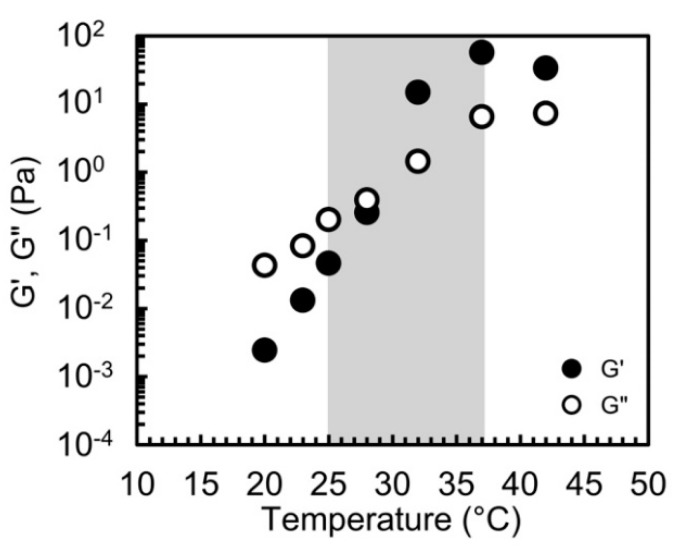
Storage and loss moduli *G*′ and *G*″ as a function of temperature of the aqueous laponite/PEG1.0k–PLGA0.8k nanocomposites with the laponite concentration of 1.0 wt % and the PEG1.0k–PLGA0.8k concentration of 2.0 wt %.

The thermo-responsive gelation behavior of the laponite/PEG-*b*-PLGA nanocomposites with PEG1.0k–PLGA0.8k was also analyzed by DMA. In particular, the specimens possessing the gelation temperature in the temperature range between 25 and 37 °C (the physiological temperature) were determined by the test-tube inversion method and selected for the DMA analysis. For example, Figure 6 shows the DMA results of the nanocomposite (2.0 wt % of PEG1.0k–PLGA0.8k and 1.0 wt % of laponite). The storage modulus *G*′ and the loss modulus *G*″ were plotted as a solid circle and an open circle, respectively. It was confirmed that as the temperature increased, *G*′ abruptly increased and eventually exceeded *G*″ around the physiological temperature. In other words, the gelation temperature was also confirmed to be around the physiological temperature by DMA analysis. In fact, the gelation temperatures of the nanocomposite (2.0 wt % of PEG1.0k–PLGA0.8k and 1.0 wt % of laponite) determined by the crossover points were 29 °C. It was also confirmed that the gelation temperatures determined by DMA matched well with the temperatures determined by the tube-inversion test. Therefore, according to the results of the tube inversion test and the DMA, it was concluded that the laponite/PEG-*b*-PLGA nanocomposites exhibited the thermos-responsive gelation behavior and possessed the gelation temperatures in the temperature range between 25 and 37 °C.

### 3.3. Microstructure of the Laponite/PEG-b-PLGA Nanocomposites

In order to investigate the mechanism of the thermo-responsive gelation behavior of the laponite/PEG-*b*-PLGA nanocomposites, the dispersion state of PEG-*b*-PLGA in the laponite/PEG-*b*-PLGA nanocomposites was analyzed by the contrast-matching SANS experiments. In particular, the contrast between laponite and water was matched in order to ignore the scattering from laponite and highlight the scattering from PEG-*b*-PLGA.

**Figure 7:**
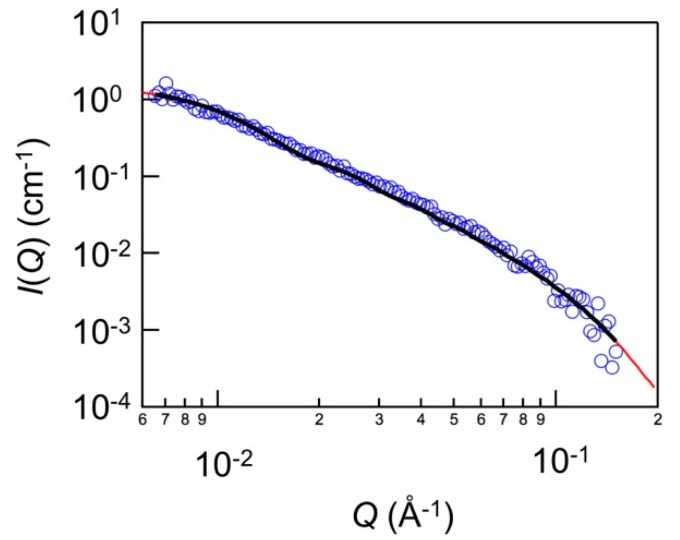
SANS profiles for the laponite dispersion in D_2_O. The solid line in the figure represents the fitting curve with Equation (1).

Prior to the contrast-matching SANS experiments of the nanocomposites, the contrast-matching point for laponite in water was determined to be at the D_2_O fraction of 66% by systematically varying the D_2_O fraction in the D_2_O/H_2_O mixture as 0, 25, 50, 75, and 100% in the same manner as described in the previous literature [22]. Figure 7 shows an example of the SANS profile of laponite in water. It was confirmed that *I*(*Q*) decreased according to the power law of *Q*^−2^ as *Q* increased. That is, the existence of the disk-shaped objects was indicated. In order to analyze the SANS profile quantitively, the model based on the form factor for the thin disks was used for fitting the SANS profile. The model is written as follows:

(1) $$P\left(Q\right)=\phi V\Delta \rho^{2}\int_{0}^{\pi /2}{\left\{\frac{2J_{1}\left(QR\sin\alpha \right)}{QR\sin\alpha }\frac{\sin\left(QH\cos\alpha \right)}{QH\cos\alpha }\right\}}^{2}\sin\alpha \;d\alpha$$

where the Bessel function *J*_1_ is given as follows:

(2) $$J_{1}=\frac{\sin x}{x}$$

where 2*H*, *R*, and *V* are the thickness, the radius, and the volume of the disk, respectively. Δ*ρ* is the difference in scattering densities between the disk and the surrounding medium. *ϕ* is the particle volume fraction. *α* is the angle between the normal vector to the disk surface and the scattering vector. The fitting result are shown as a solid line in Figure 7. The radius and the thickness of the laponite used in this study was found to be ~23 nm and ~2.7 nm, respectively. The calculated value was slightly larger than the reported values in the previous study [22,25]. This could be due to the aggregation of laponite platelets depending on the preparation method of the laponite suspensions.

**Figure 8:**
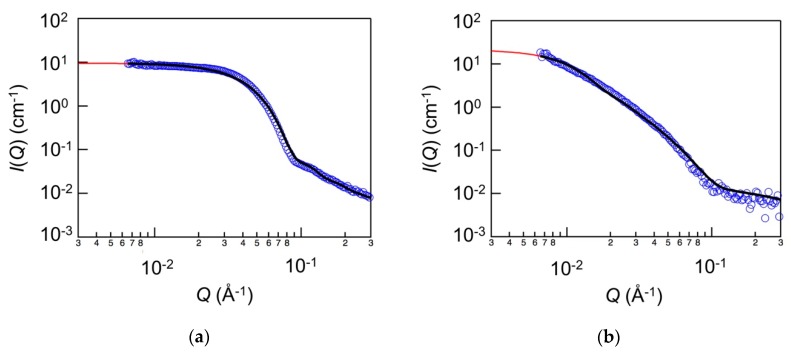
SANS profiles of PEG-*b*-PLGA (**a**) in single-body solution and (**b**) in the laponite/PEG-*b*-PLGA nanocomposite. The solid line in the figure represents the fitting curve with Equations (3), (5), and (6).

Figure 8 shows the SANS profile of PEG-*b*-PLGA diblock copolymers in the single-body PEG-*b*-PLGA solution (Figure 8a) and in the laponite/PEG-*b*-PLGA nanocomposites (Figure 8b). The results with PEG1.0k–PLGA0.8k are shown representatively. As shown in Figure 8a, *I*(*Q*) decreased according to the power law of *Q*^−4^ as *Q* increased. That is, the existence of the spherical objects was indicated. In order to analyze the SANS profile quantitively, the model based on the core-shell spherical model was used for fitting the SANS profile. The model is written as follows [23]:

(3) $$P\left(Q\right)=\frac{\phi }{V_{s}}{\left[\frac{3V_{c}\left(\rho_{c}-\rho_{s}\right)J_{1}\left(QR_{C}\right)}{QR_{C}}+\frac{3V_{s}\left(\rho_{s}-\rho_{solv}\right)J_{1}\left(QR_{s}\right)}{QR_{s}}\right]}^{2}$$

where the Bessel function *J*_1_ is given as follows:

(4) $$J_{1}=\frac{\sin x-x\cos x}{x^{2}}$$

where *V*_s_ is the particle volume including the outer shell, *V*_c_ is the volume of the core, *R*_s_ is the radius of the particle including the outer shell, and the *R*_c_ is the radius of the core, *ρ*_s_ is the scattering densities of the shell, *ρ*_c_ is the scattering densities of the core, *ρ*_solv_ is the scattering densities of the solvent. *ϕ* is the particle volume fraction. Additionally, for the fitting in the higher *Q* region, the Debye function was also included. This was because, in the previous study, it was reported that the scattering profile of the polymer micelle systems could not be described only by the core-shell model due to the occurrence of the blob scattering from the micelle shell [26]. The Debye function is written as follows:

(5) $$P\left(Q\right)=\phi V\Delta \rho^{2}2\frac{e^{-{\left(qQ\right)}^{2}}+{\left(QR_{g}\right)}^{2}-1}{{\left(QR_{g}\right)}^{4}}$$

where *ϕ* is the volume fraction of polymer, *V* is the volume of polymer per chain, Δ*ρ* is the difference in the scattering densities between the polymer and the solvent, *R*_g_ is the radius of gyration. The fitting results are shown as a solid line in Figure 8a. It was confirmed that the SANS profile was well described by the sum of the core-shell model and the Debye function. The radius of micelles and the radius of micellar core were found to be ~4.7 nm and ~1.6 nm. The measured values show good agreement with the values presented in the previous studies, considering the difference in the molecular weight [23]. On the other hand, as shown in Figure 8b, for the PEG-*b*-PLGA in the laponite/PEG-PLGA nanocomposites, *I(Q)* decreased according to the power law of around *Q*^−2^ as *Q* increased. It was inferred that the adsorption of PEG-*b*-PLGA on the laponite surface was induced and the micellar structure of PEG-*b*-PLGA changed according to the shape of laponite working as a template. In order to analyze the SANS profile quantitively, the model based on the oblate core-shell ellipsoid model was used for fitting the SANS profile. The model is written as follows [23,24]:

(6) $$P\left(Q\right)=\frac{\phi }{V_{s}}\int_{0}^{1}{\left|F\left(Q,\;R,\;\alpha \right)\right|}^{2}d\alpha$$

where the *F*(*Q*, *R*, *α*) is given as follows:

(7) $$F\left(Q,\;R,\;\alpha \right)=\frac{3V_{c}\left(\rho_{c}-\rho_{s}\right)J_{1}\left(u_{core}\right)}{u_{core}}+\frac{3V_{s}\left(\rho_{s}-\rho_{solv}\right)J_{1}\left(u_{shell}\right)}{u_{shell}}$$

where the *u* is given as follows:

(8) $$u=Q\sqrt{R_{maj}{}^{2}\left(1-\alpha^{2}\right)+R_{min}{}^{2}\alpha^{2}}$$

where *V*_s_ is the particle volume including the outer shell, *V*_c_ is the volume of the core, *R*_maj_ and *R*_min_ are the major and minor radius, *ρ*_s_ is the scattering densities of the shell, *ρ*_c_ is the scattering densities of the core, and *ρ*_solv_ is the scattering densities of the solvent. *ϕ* is the particle volume fraction. *J*_1_ is the Bessel function given as Equation (4). As well as in Figure 8a, for the fitting in the higher *Q* region, the Debye function described as Equation (4) was included. The fitting result are shown as a solid line in Figure 8b. It was confirmed that the SANS profile was well described by the sum of the oblate core-shell ellipsoid model and the Debye function. The major and minor radius of micelles were found to be ~25 and ~3.2 nm.

## 4. Discussion

From all these results, the thermo-responsive gelation could be successfully achieved using PEG-*b*-PLGA diblock copolymers showing high solubility in water, and the *T*_gel_ was neatly regulated by the concentration of PEG-*b*-PLGA and laponite. In fact, the thermo-responsive gelation around the physiological temperature could be achieved at the minimum solute concentration of only 2.75 wt % (i.e., the total concentration of laponite (0.75 wt %) and PEG-*b*-PLGA (2 wt %)). As compared to the previous research using the nanocomposite approach, the *T*_gel_ of the composites based on Pluronic F127 and laponite was around 70 °C at the solute concentration of ~2.6% (i.e., the total concentration of laponite (1.2 wt %) and F127 (1.4 wt %)) [9]. The reported *T*_gel_ was exceedingly higher than the physiological temperature, while the critical concentration for the thermo-responsive gelation was comparable with the concentration reported in this study. As for the single-body solution of block copolymers based on PEG and PLGA, the PEG-*b*-PLGA solution generally presents the sol state in the wide concentration range. The solution of PEG-*b*-PLGA-*b*-PEG triblock copolymers forming “star-like” micelles also presents the sol state in the wide concentration range. In fact, in the previous study, it was reported that, the PEG-*b*-PLGA-*b*-PEG concentration presenting thermo-responsive gelation with the gel state at 37 °C was relatively as high as 28 wt % for the single-body solution of the PEG-*b*-PLGA-*b*-PEG with the total PEG molecular weight of 1100 g/mol and with the PEG/PLGA ratio of ~0.5 [2,4]. Therefore, the advantage of the approach reported in this paper is the achievement of the thermo-responsive gelation around the physiological temperature using PEG-*b*-PLGA at lower solute concentration. The findings could strongly support the potential of the laponite/PEG-PLGA nanocomposite for the pharmaceutical and biomedical applications. This is because the nanocomposites with laponite were reported to exhibit the potential for the biomedical applications as described in the previous papers [27,28].

On the other hand, as for the laponite, the laponite suspension presents the gel state at the concentration of 2 wt % or higher. The critical gelation concentration required for the single-body laponite suspension is much closer to the critical gelation concentration of the laponite/PEG-*b*-PLGA nanocomposites (2.75 wt %). Therefore, it was inferred that the preformed laponite networks existing in water, and that the PEG-*b*-PLGA diblock copolymers worked as thermo-responsive agents and helped the preformed laponite networks connect in response to the temperature increase.

As for the gelation temperature, the *T*_gel_ of the laponite/PEG-*b*-PLGA nanocomposites increased as the PEG/PLGA ratio increased, and the *T*_gel_ of the laponite/PEG-*b*-PLGA nanocomposites decreased as the laponite concentration increased. The results could also support the infer mentioned above. In the previous literature, the degree of micellar aggregation in aqueous amphiphilic copolymer solutions was proposed as the key element for *T*_gel_. In order to obtain the sufficient degree of micellar aggregation for the gelation, the PEG1.0k–PLGA0.8k with the shorter hydrophobic block should be at relatively higher temperature due to the less hydrophobicity of the copolymers, as compared to the PEG1.0k–PLGA1.6k. On the other hand, the network formation of the laponite was induced and the gel state became dominant even at low temperature with the increase of the laponite concentration.

Considering the results of microstructural analysis and the fact that the micellar structure of PEG-*b*-PLGA in the composites resembled the shape of laponite and the major radius of PEG-*b*-PLGA micelles in the nanocomposite was close to the radius of laponite, it was suggested that PEG-*b*-PLGA diblock copolymers were on the surface of the laponite platelets due to the nature of polymeric surfactants. In the previous studies, the absorption of surfactants on laponite has been reported [25,29,30,31,32,33,34]. In these reports, it was reported that the hydrophobic block preferred to locate at the surface of laponite. Therefore, the interaction between PEG-*b*-PLGA and laponite could also be the hydrophobic interaction.

To wrap up the results in this study, the thermo-responsive sol-gel transition of the nanocomposite could be explained as follows: The laponite formed the house-carded network or the aggregates during the preparation of the laponite suspension. After mixing the PEG-*b*-PLGA solution and the laponite suspension, the preformed laponite network or the aggregates of the laponite platelets could be covered with PEG-*b*-PLGA diblock copolymers due to the nature of polymeric surfactants. Then, once the solution was heated, the preformed laponite network or the aggregates of the laponite platelets covered with thermo-responsive PEG-*b*-PLGA diblock copolymers could be connected by the thermo-responsive hydrophobic physical aggregations of the copolymers, leading to the formation of the inhomogeneous networks, eventually causing the sol-gel transitions.

In this paper, it was revealed that the thermo-responsive hydrogels could be obtained through the nanocomposite approach by using highly water-soluble diblock copolymers known as polymeric surfactants. Additionally, since both PEG-*b*-PLGA diblock copolymer and laponite are widely-studied as prospective candidates for biomedical application, the laponite/PEG-*b*-PLGA nanocomposites could possess the potential for the biomedical application. In order to thoroughly understand the behavior, more detailed analyses such as the physicochemical characterization and the temperature-dependence and concentration-dependence microstructural analysis would be required. Considering the application of the laponite/PEG-b-PLGA nanocomposites, biomedical tests would be also required.

## 5. Conclusions

The PEG-*b*-PLGA diblock copolymers were found to work as thermo-responsive gelling agents by adding the synthesized PEG-*b*-PLGA diblock copolymers to the aqueous laponite suspension. The PEG-*b*-PLGA diblock copolymer is generally known as a polymeric surfactant for drug delivery and was not used as a gelling agent. In detail, PEG-*b*-PLGA diblock copolymer with high solubility in water (i.e., with high PEG/PLGA ratio of 1.3) was synthesized for thermo-responsive nanocomposite hydrogels. In the preliminary experiment, it was confirmed that the synthesized PEG-*b*-PLGA diblock copolymers exhibited high solubility in water due to the high PEG/PLGA ratio. It was found that the solute concentration was relatively low (PEG-*b*-PLGA: 2–5 wt % and laponite: 0.75–1.25 wt %). It was also found that the *T*_gel_ could be regulated to be around the physiological temperature (25–37 °C) by controlling the solute concentration. In more detail, the *T*_gel_ of the laponite/PEG-*b*-PLGA nanocomposites increased as the PEG/PLGA ratio increased, and the *T*_gel_ of the laponite/PEG-*b*-PLGA nanocomposites decreased as the laponite concentration increased. Finally, PEG-*b*-PLGA diblock copolymers were also confirmed to be on the surface of the laponite platelets due to the nature of polymeric surfactants, and the thermosensitive PEG-*b*-PLGA diblock copolymers on the laponite surface could trigger the thermo-responsive connection of the preformed laponite network. From all these findings, the thermo-responsive gelation behavior of the laponite/PEG-*b*-PLGA nanocomposite was understood and the mechanism of the thermo-responsive gelation was suggested.

## Figures and Tables

**Table 1:** Characteristics of PEG and PEG-*b*-PLGA diblock copolymers.

Sample Name	*M*_n_, _PEG_	*M*_n_, _PLGA_^1^	PEG/PLGA	LA/GA ^1^	*M*_n_, _total_^2^	*M*_w_/*M*_n_^2^
PEG1.0k	1000	-	-	-	1374	1.06
PEG1.0k–PLGA0.8k	1000	785	1.27	1.6	2867	1.14
PEG1.0k–PLGA1.6k	1000	1596	0.63	1.9	3768	1.24

^1^ Calculated from ^1^H-NMR spectrum. ^2^ Measured by GPC using polystyrene standards.
